# The Application of Mixed Organic and Inorganic Fertilizers Drives Soil Nutrient and Bacterial Community Changes in Teak Plantations

**DOI:** 10.3390/microorganisms10050958

**Published:** 2022-05-02

**Authors:** Qingqing Zhang, Weiwei Zhao, Zaizhi Zhou, Guihua Huang, Xianbang Wang, Qiang Han, Gaofeng Liu

**Affiliations:** Research Institute of Tropical Forestry, Chinese Academy of Forestry, Guangzhou 510520, China; qqzt19@caf.ac.cn (Q.Z.); zhao_vivi@163.com (W.Z.); huanggh@ritf.ac.cn (G.H.); wangxb@caf.ac.cn (X.W.); hanqiang1988@163.com (Q.H.); yllgf2006@126.com (G.L.)

**Keywords:** *Tectona grandis*, soil pH, bacterial abundance, dominant phylum, co-occurrence network, mixed fertilization

## Abstract

Appropriate fertilization can enhance forest productivity by maintaining soil fertility and improving the structure of the bacterial community. However, there is still uncertainty surrounding the effects of combined application of organic and inorganic fertilizers on soil nutrient status and bacterial community structure. A fertilization experiment was set up in an eight-year-old teak plantation with five treatments involved: mixed organic and NPK compound fertilizers (OCF), mixed organic and phosphorus fertilizers (OPF), mixed organic, NPK and phosphorus fertilizers (OCPF), mixed NPK and phosphorus fertilizers (CPF) and no fertilization (CK). Soil chemical properties and bacterial communities were investigated, and the co-occurrence pattern of the bacterial community under different fertilization treatments was compared. The results showed that the contents of soil organic matter and nitrate nitrogen, and the soil pH values were the highest after OCPF treatment, which were 20.39%, 90.91% and 8.16% higher than CK, respectively. The richness and diversity of bacteria underwent no obvious changes, but the structure of the soil’s bacterial community was significantly altered by fertilization. Of the dominant bacteria taxa, the relative abundance increased for Gemmatimonadetes, Myxococcota, ADurb.Bin063-13 and Candidatus_Koribacter, and decreased for Chloroflexi, Proteobacteria, JG30-KF-AS9 and Acidothermus under OCPF treatment in comparison to CK. The number of nodes and edges, the average degree and the network density of bacterial community co-occurrence networks were the greatest in OCPF treatment, indicating that application of OCPF could make the network structure of soil bacteria more stable and complex. Moreover, soil pH and organic matter were significantly correlated with bacterial community structure and were considered the main influencing factors. These findings highlight that the combined application of organic, NPK and phosphorus fertilizers is highly beneficial for improving soil quality and optimizing bacterial community structure in teak plantations.

## 1. Introduction

Fertilization is one of the pivotal management measures in the timber forest cultivation process. It directly affects soil fertility and forest productivity, as well as the diversity and composition of microorganisms by changing soil aggregate structure and nutrient levels [1,2]. Applying inorganic fertilizers is regarded as the fastest and most direct way to effectively increase soil nutrient content and accelerate tree growth and timber output. However, it can also generate negative impacts on soil health, such as soil acidification and degradation [3,4]. Likewise, the soil microbial community is also highly sensitive to the addition of inorganic fertilizers. Observable consequences seem to be the decreasing population and biodiversity of microorganisms, as well as instability of the community structure [5,6,7]. In recent years, the application of organic fertilizers for soil property has been greatly emphasized [8,9]. Some studies have shown that the application of organic fertilizers could significantly enhance soil quality in terms of soil structure, physicochemical properties and biological characteristics [10,11]. The combined application of organic and inorganic fertilizers could mediate organic carbon (C) sequestration, as well as elevate the nitrogen (N) and phosphorus (P) balance in soil environments [12]. Moreover, the addition of organic fertilizers is the key to relieving soil acidification [13,14].

Soil microorganisms are an extremely abundant biotic group and are important for maintaining the diversity and stability of the terrestrial ecosystem [15,16]. Bacteria play a decisive role in forest soil environments by participating in biogeochemical cycles, such as litter and dead root decomposition and nitrogen and phosphorus mineralization [17]. As the major biological regulator of nutrient cycling [18], bacteria groups are also very sensitive to changes in the soil environment, especially with the increase of available nutrients due to external inputs. However, it is widely acknowledged that mineral fertilizers generally decrease the diversity of soil bacterial communities [7,19] and may potentially decimate the ecosystem services provided by microorganisms [20,21]. In contrast, organic fertilizers directly provide labile carbon and a large number of energy substances, stimulating the activity of soil microorganisms [22]. The soil after the combined application of organic and inorganic fertilizers exhibited more abundant bacteria and a higher level of biodiversity [14]. The survival strategies of microbes also shifted to adapt to the changes in the soil environment after organic fertilization; in particular, the abundance of beneficial bacteria associated with the cycling of carbon, nitrogen and phosphorus in soil increased substantially [8,23]. For example, species of Firmicutes and Proteobacteria bacterial phyla prefer nutrient-rich environments and are enriched in the soil after organic fertilization [24]. Ye et al. also found that the addition of biofertilizers enriched the Actinobacteria phylum that was closely related to the decomposition of complex organic compounds [25]. However, some scholars considered that the diversity or richness had no notable changes after organic fertilizers [8,26], and the relative abundance of Firmicutes and Proteobacteria declined [27]. The response of microbes to fertilizers varies according to a series of factors and can be highly related to soil heterogeneity. Hence, more research is needed to understand the underlying changes in soil microorganisms after fertilization.

Bacterial communities are characterized not only by the composition and number of species, but also by the interactions and associations among different taxa [28]. The bacterial co-occurrence network was performed to reveal the interrelationships between species and the complexity of community structure and function [29,30,31]. Previous studies confirmed that alterations of community structure and bacterial network were closely related to soil properties, such as pH [32], soil matter organic [33,34] and available phosphorus [35], which have been considered major drivers of variation in biotopes. Although symbiotic network patterns were universally applied in forest ecosystems, the response of complex bacterial communities to the application of mixed organic and inorganic fertilizers in teak plantations is still unknown.

Teak (*Tectona grandis* L.f.) is one of the most precious hardwood species in the tropics, known for its excellent timber quality, and it is used extensively in the manufacture of high-grade furniture and wooden floors [36]. Fertilization is widely conducted in the practice of teak plantation forestry. Studies have shown that the application of chemical fertilizer can promote nitrogen and phosphorus absorption in trees and accelerate stem growth [37,38,39]. The addition of organic fertilizer can also promote the diameter growth of teak and improve the soil quality of its plantations [40]. There was little focus on the response of soil nutrients and bacterial communities to the combined application of organic−inorganic fertilizers in teak plantations. Thus, the objectives in the present study are to analyze the differences in soil chemical characteristics and bacterial community composition between different fertilization treatments, and to identify the key factors influencing the structure of bacterial communities.

## 2. Materials and Methods

### 2.1. Study Site

The study site is located in Luodian county, Qiannan Prefecture, Guizhou Province, China (106°46′ E, 25°25′ N, elevation 413 m to 449 m). This region has a subtropical monsoon humid climate with a mean annual temperature of 19.6 °C. The average annual rainfall is about 1400 mm. The daylight hours and annual frost-free period are 1350–1520 h and 335 days, respectively. The soil was identified as yellow−red earth and derived from sand shale. Teak seedlings of the fine clone 7544 were used for afforestation, which was received from the Research Institute of Tropical Forestry, Chinese Academy of Forestry in Guangzhou, China. The experimental plantation was planted in 2010 at a spacing of 2.5 m × 3.0 m.

### 2.2. Experimental Design

The fertilization experiment was established in a randomized block design and included five treatments: the combined application of organic and NPK compound fertilizers (OCF); the organic and CaMgP fertilizers (OPF); the mixed application of organic, NPK and CaMgP fertilizers (OCPF); the combined application of NPK and CaMgP fertilizers (CPF); and no fertilization as a control (CK). Each treatment was repeated four times. A total of 20 treatment plots of 270 m^2^ each were set up. Fertilization was conducted in May 2018 and 2019, and annual fertilizer inputs are listed in Table S1.

The commercial organic fertilizer includes 45% organic matter content and more than 5% NPK content. NPK compound fertilizer is composed of N, P_2_O_5_ and K_2_SO_4_ in the ratio 14:16:15. CaMgP fertilizer contains 18% of P_2_O_5_, 45% of CaO and 12% of MgO. All inorganic and organic fertilizers were obtained from Hubei Xinyangfeng Fertilizer Co., Ltd., Jingmen, China.

### 2.3. Soil Sampling and Chemical Analysis

Soil samples of the surface layer (0–10 cm) were collected two years and four months after the final fertilization (in September 2021). Five sampling points in each plot were randomly selected, and soil samples taken from the five points were mixed together evenly. First, the plant roots, residues and break stones in the soil samples were removed. The samples were then passed through a 2 mm mesh sieve and divided into two sections: one was stored at −80 °C in a refrigerator for DNA extraction, and the other was used for the determination of soil chemical properties.

The pH value of each soil sample was determined with an acidimeter (PHS-3C, Shanghai, China) in a solution that was mixed with double-distilled water and soil (2.5:1). The content of soil organic matter (SOM) was measured using the potassium dichromate oxidation method [41]. Ammonium nitrogen (NH_4_^+^–N) was assayed using a spectrophotometric method, with Nassi reagent acting as the extraction agent. Nitrate (NO_3_—N) in the soil was extracted with 2M KCl and measured by ultraviolet spectrophotometry. Available phosphorus (AP) content was extracted from soil samples with a mixed solution of ammonium fluoride and hydrochloric acid and determined using the visible spectrophotometer [42,43].

### 2.4. DNA Extraction and PCR Amplification

DNA extractions from 0.5 g fresh soil samples were performed using the Magnetic soil DNA kit (Tiangen Biotech Co., Ltd., Beijing, China), referring to the manufacturer’s instructions. The extracted DNA concentration and purity were monitored on 1% agarose gels, and the concentration was diluted to 1 ng·μL^−1^ with sterile water to ensure amplification efficiency and accuracy. The V3–V4 hypervariable regions of bacterial 16S rRNA genes were amplified by polymerase chain reaction (PCR) with the forward primer (341F 5′-CCTAYGGGRBGCASCAG-3′) and the reverse primer (806R 5′-GGACTACNNGGGTATCTAAT-3′). The PCR amplification mixture contained 15 μL Phusion^®^ High-Fidelity PCR Master Mix (New England Biolabs), 0.2 μM forward primer, 0.2 μM reverse primer and 10 ng template DNA. The thermal cycling program of the PCR reaction process included an initial denaturation step at 98 °C for 1 min, followed by 30 cycles of denaturation at 98 °C for 10 s, annealing at 50 °C for 30 s, extension at 72 °C for 30 s, and then a final 5-min extension at 72 °C. PCR reactions were subjected to three replicates [28,44].

### 2.5. Illumina NovaSeq Sequencing and Bioinformatics Analysis

PCR products were mixed with an equal volume of 1 X loading buffer and detected by electrophoresis on 2% agarose gel, which was then purified using a Qiagen Gel Extraction Kit (Qiagen Inc., Frank furt, Germany). NEBNext^®^ Ultra™ IIDNA Library Prep Kit (Cat No. E7645) was used to sequence the construction of libraries following the manufacturer’s protocols, and the library quality was evaluated on the Qubit^®^ 2.0 Fluorometer (Thermo Fisher Scientific Inc., Waltham, MA, USA). Finally, the library was paired-end sequenced on an Illumina Novaseq platform (Illumina, San Diego, CA, USA) according to the standard protocols of Novogene Co., Ltd. (Beijing, China). The raw data have been stored in the Sequence Read Archive (SRA) data of the National Center for Biotechnology Information (NCBI) database with accession number PRJNA820355.

For the raw reads, quality filtering was done using the fastp (Version 0.20.0) software and merged using FLASH (Version 1.2.11 http://ccb.jhu.edu/software/FLASH/ 27 March 2022) [45]. Then, the chimera sequences were detected and removed using Vsearch (Version 2.15.0) [46]. Amplicon Sequence Variants (ASVs) were obtained by denoising with DADA2 and filtering out ASVs with abundance less than 5 [47]. ASV taxonomy based on classify-sklearn with the Sliva Database (https://www.arb-silva.de/ 27 March 2022) and alpha diversity indexes were calculated in QIIME2 software (Version QIIME2-202006).

### 2.6. Statistical Analysis

One-way analysis of variance (ANOVA) and Tukey’s honestly significant difference (HSD) test in SPSS25.0 software (SPSS Inc., Chicago, USA) was used to determine the differences in soil chemical properties and alpha-diversity indexes that were normalized by using a log_10_ (X + 1) conversion. Significant differences in the relative abundance of dominant bacteria at phylum and genus levels were analyzed by the Kruskal−Wallis test. Principal Coordinate Analysis (PCoA) of weighted unifrac distances was used to explore the beta-diversity changes between bacterial communities for each sample. Analysis of Similarities (ANOSIM, based on Weighted Unifrac distances) by Anosim function within QIIME2 software was used to test community structure discrepancies among different treatments. Linear discriminant analysis (LDA) and effect size (LEfSe) analysis were conducted to identify potential bacterial biomarkers of each treatment. Mantel tests (vegan package in R software, http://www.r-project.org/ 27 March 2022) were used to test the relationship between the soil environment and the distribution of bacterial communities (at the ASV level). Redundancy analysis (Canoco 5.0 software, http://www.microcomputerpower.com/ 27 March 2022) and Spearman’s correlation analysis were applied to evaluate the influence of edaphic factors on the main bacterial phyla and genera.

Co-occurrence networks were used to explore the interactions between soil bacterial taxa (based on ASV levels). The network construction was based on Pearson’s correlation analysis by using the psych package in Rstudio software (http://www.rstudio.com/ 27 March 2022) and the correlation coefficient and significance level (*R* > 0.06, *p* < 0.05) was set up. Analytical data were the ASVs with the top 100 absolute abundance in each sample [48,49]. Visualization of network relationships and calculation of topological properties were done using Gephi0.9.2 software (http//:gephi.org/ 27 March 2022).

## 3. Results

### 3.1. Changes in Soil Chemical Properties in Teak Plantations

The combined fertilization enhanced the soil pH value and SOM content (Table 1, *p* < 0.05). The soil pH value in OCPF treatment was significantly higher than CK by 8.16%, and the SOM content also increased by 20.39% and 17.53% compared to CK and CPF, respectively. The average content of NH_4_^+^–N and NO_3_^−^–N in OCF, OCPF and CPF treatments were noticeably greater than those in CK. The content of NH_4_^+^–N and NO_3_^−^–N in OCF treatment were 152.94% and 90.59% greater than CK, respectively. The soil AP content showed an increasing trend with increased fertilization in the order: OCF > CPF > OPF > OCPF > CK.

### 3.2. Sequence Characteristics and Soil Bacterial Community Diversity

**Figure 1:**
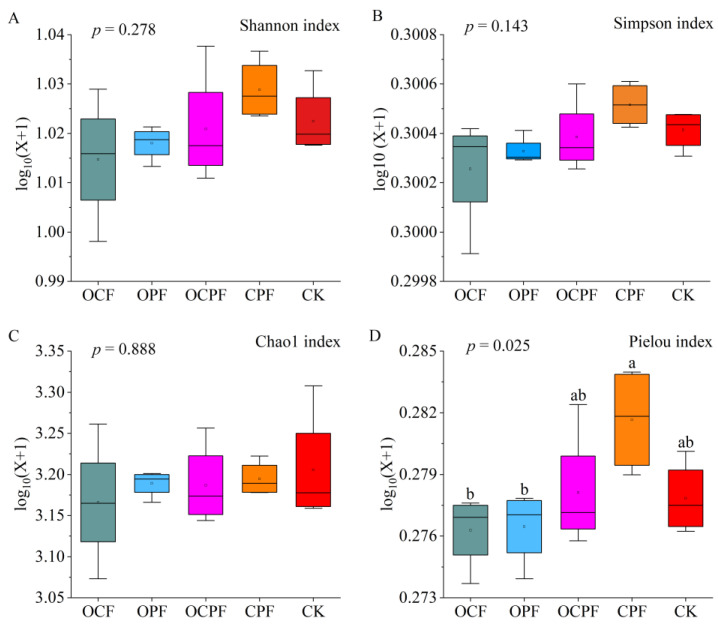
Alpha diversity indexes (*n* = 4) of bacterial communities between different fertilization treatments. (**A**), Shannon index; (**B**), Simpson index; (**C**), Chao1 index; (**D**), Pielou index. OCF, application of organic fertilizer and NPK compound fertilizer; OPF, application of organic and CaMgP fertilizers; OCPF, application of organic, NPK and CaMgP fertilizers; CPF, application of NPK and CaMgP fertilizers; CK, no fertilization. Lowercase letters indicate statistical differences at a 0.05 significance level among treatments.

A total of 2,215,833 reads were obtained after sequencing all soil samples, and after denoising and filtering, 1,266,203 effective sequences were used for subsequent bacterial community analysis. The coverage index (99.95–100%) showed that the sequencing depth effectively reflected the actual richness of soil bacterial communities (Table S1). The average quantity of effective sequences decreased after fertilization when compared with no fertilization. The mean length of effective sequences under CPF was the longest, and was significantly higher than that of CK, OCF and OPF treatments (Table S2). A total of 10,048 ASVs were retained in 20 samples. The Venn diagrams showed that 837 bacterial ASVs were shared among different treatments and 993 ASVs were shared between four fertilization treatments. The number of specific ASVs in CPF and OCPF treatments was greater than in CK (Figure S1). Based on the Shannon, Simpson and Chao1 indexes, no significant differences in soil bacterial diversity and richness were found between different fertilization treatments (Figure 1A–C). However, the Pielou’s evenness index in CPF and OCPF treatments was significantly higher than in OCF and OPF treatments (Figure 1D).

### 3.3. Impact of Fertilization on Bacterial Community Structure

The community structure of soil bacteria, based on the ANOSIM analysis, varied significantly among different fertilization treatments (*R* = 0.372, *p* = 0.005). The PCoA analysis explained 60.94% of the total variation in bacterial communities, of which PC1 accounted for 42.32%. However, soil bacterial communities were not clearly clustered into different groups according to the different fertilization treatments in the ordination diagram (Figure S2).

**Figure 2:**
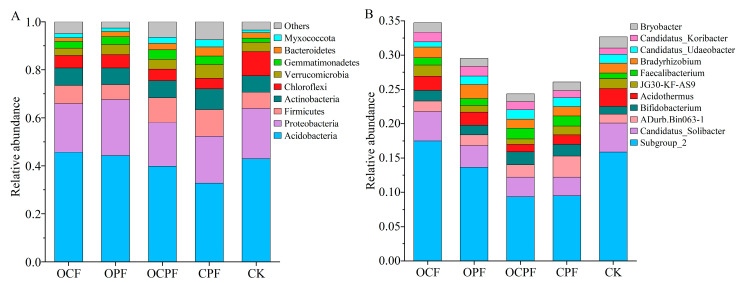
The relative abundance (>1%) of dominant bacteria at the phylum (**A**) and genus (**B**) levels. OCF, application of organic fertilizer and NPK compound fertilizer; OPF, application of organic and CaMgP fertilizers; OCPF, application of organic, NPK and CaMgP fertilizers; CPF, application of NPK and CaMgP fertilizers; CK, no fertilization.

A total of 36 bacteria phyla and 619 genera with definite groups were detected by species annotation of all ASVs. The mean relative abundance of 9 phyla and 11 genera exceeded 1% (Figure 2B). At the level of phylum, the relative abundance of Acidobacteria (32.79–45.64%) was the highest, followed by Proteobacteria (18.24–23.27), Firmicutes (6.28–10.29%), Actinobacteria (6.97–8.68%) and Chloroflexi (4.33–10.10%). Other bacterial phyla occupied only a minor proportion (Figure 2, Table S4).

**Figure 3:**
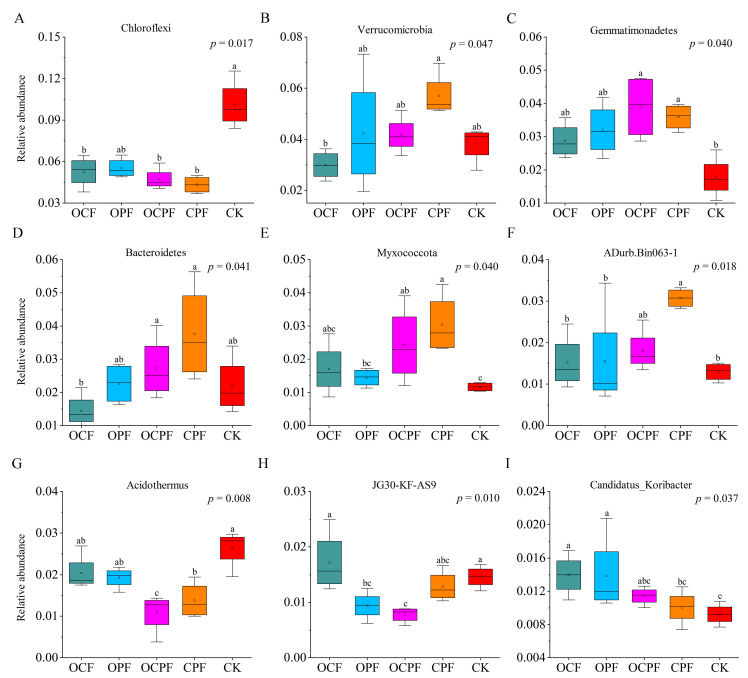
Changes in bacterial community composition among different treatments at the dominant phylum (**A**–**E**) and genus (**F**–**I**) level. Significant differences are shown by different lowercase letters at a significance level of 0.05 according to the Kruskal−Wallis test.

The relative abundance significantly declined for Chloroflexi, while it increased for Gemmatimonadetes and Myxococcota under OCPF and CPF treatments, compared to CK (Figure 3, *p* < 0.05). Verrucomicrobia and Bacteroidetes (except for OCF) showed higher relative abundances in fertilization treatments than those in the CK group. The relative abundance of Firmicutes and Actinobacteria was increased in OCP, OCPF and CPF treatments, whereas Acidobacteria and Proteobacteria in OCPF and CPF treatments were less abundant than that of CK (Table S4). At the genus level, Subgroup_2, Candidatus_Solibacter and ADurb.Bin063-1 were the three most dominant bacterial genera (Figure 2B). The relative abundance of ADurb.Bin063-1, Acidothermus, Candidatus_Koribacter and JG30-KF-AS9 presented significant differences among the fertilization treatments. Candidatus_Koribacter showed a prominent increase in OCF and OPF but a decrease for Acidothermus in comparison to CK. Application of OCPF markedly reduced the relative abundance of Acidothermus and JG30-KF-AS9 compared to CK (Figure 3, Table S4).

### 3.4. Biomarker Taxa of Soil Bacterial Communities

**Figure 4:**
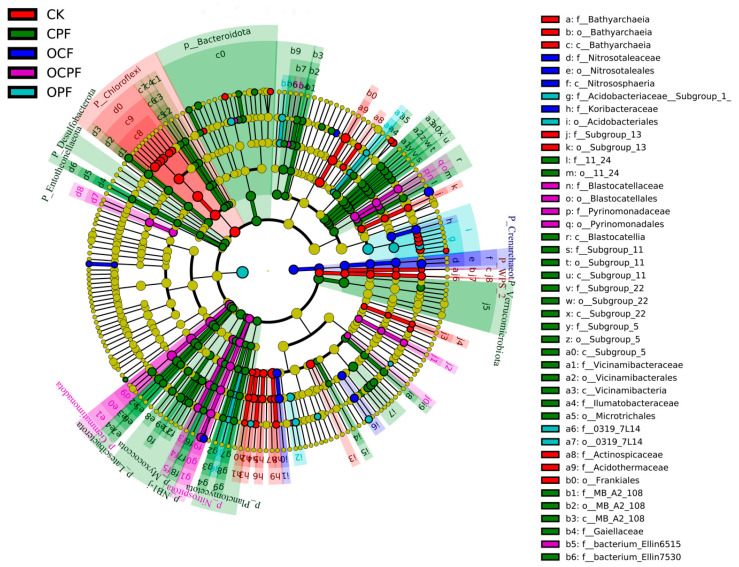
LEfSe analysis of soil bacterial biomarkers after different fertilization treatments. The circles radiating from the innermost to the outermost represent the taxonomic levels from phylum to species in the cladogram. The size of the circles is proportional to the size of the relative abundance. Species with no significant differences are colored uniformly yellow, and differential species biomarkers follow the group for coloring.

A total of 218 biomarkers of 17 phyla were identified in all treatments (Figure 4 and Figure S3). There were 28 taxa in OCPF, with the major enriched taxa to OCPF being Gemmatimonadetes and Nitrospirae (Figure 4). 18 taxa were significantly enriched in OPF treatment, and Acidobacteriales (order) had the highest relative abundance. The number of biomarkers enriched in OCF treatment was minimal, and the main significant groups were Crenarchaeota and Acidobacteria. There were more biomarkers in CK and CPF than in other treatments, and Chloroflexi (Ktedonobacterales) in CK and Verrucomicrobia and Bacteroidetes in CPF were more abundant (Figure S3).

### 3.5. Co-Occurrence Network of Bacterial Communities

**Figure 5:**
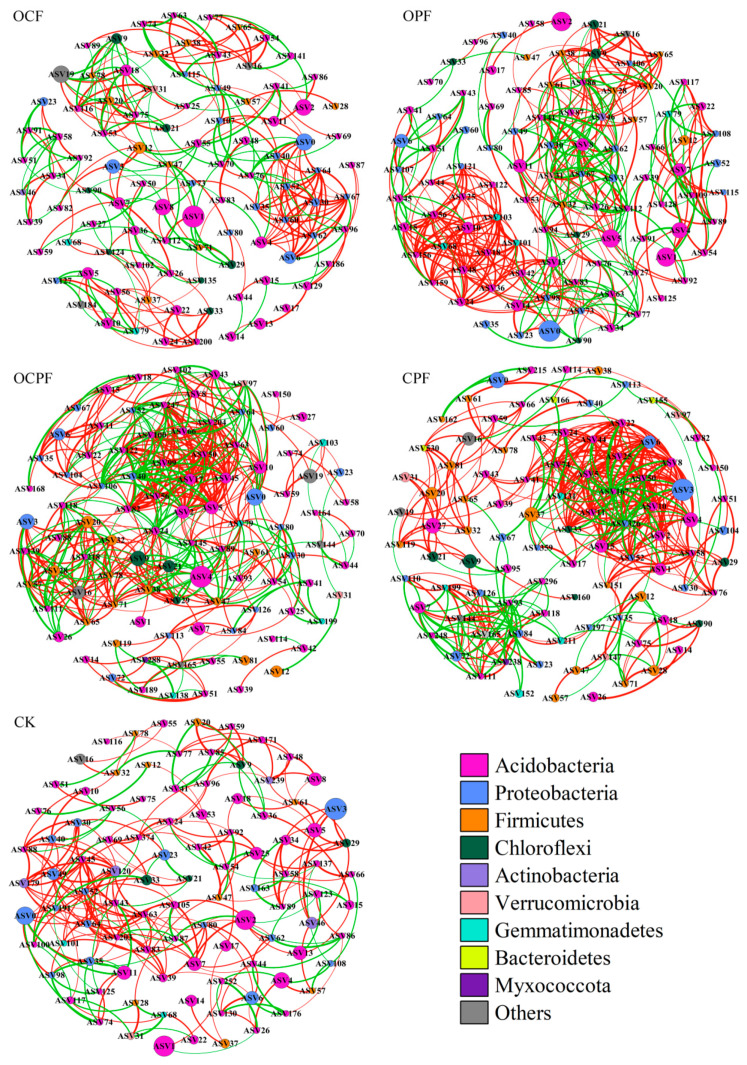
The co-occurrence network of bacterial communities at the ASV level in different fertilization treatments. The nodes in the network represented the 100 most abundant ASVs and each node size is based on their relative abundance. The red and green edges represented the positive and negative correlation between bacteria, respectively (Pearson, *p* < 0.05). The thickness of edges mainly depends on the correlation coefficient.

Co-occurrence networks were built based on Pearson’s correlation analysis of soil bacterial communities. The topological attributes showed that fertilization changed the interactions among bacterial taxa (Figure 5; Table 2). The number of edges in OCF, OPF, OCPF, CPF and CK was 285, 431, 500, 426 and 273, respectively. The proportion of positive edges tended to decrease and negative edges increased after fertilization (Table 2). The network density and average degree also increased and reached the maximum in OCPF treatment, indicating that the application of OCPF increased the complexity of the bacterial community. In contrast, the number of network edges in OCF and CK was lower than in the others, showing an obvious modularization and a longer average path length. The hubs (higher connect nodes and edges) in OCPF were ASV204, ASV106 and ASV79, which belonged to Acidobacteria, Proteobacteria and Gemmatimonadetes, respectively. Acidobacteria and Proteobacteria in CPF and CK, Proteobacteria and Firmicutes in OCF, Acidobacteria, Proteobacteria and Gemmatimonadetes in OPF were the central taxa in the corresponding soil (Figure 5).

### 3.6. Relationships between Soil Factors and Bacterial Community Structure

**Figure 6:**
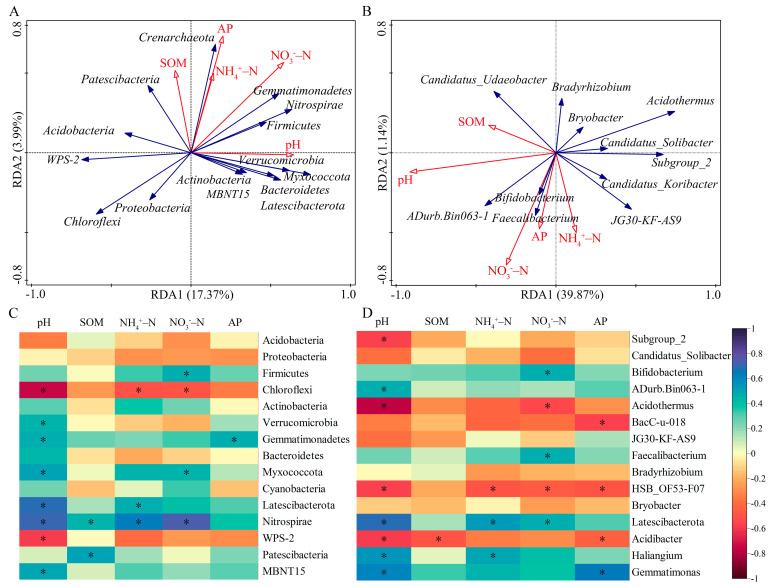
Redundancy analysis of bacteria phylum (**A**), genus (**B**) and soil factors. Spearman’s correlation analysis between bacterial species at the phylum (**C**) and genus (**D**) levels and soil chemical properties. * *p* < 0.05.

The Mantel tests suggested that soil pH and SOM have a strong effect on the structure of the bacterial community (Table S5, *p* < 0.05). Redundancy analysis between the relative abundance of bacteria and soil factors demonstrated that the soil chemical properties explained 21.36% and 40.23% of the total variation on phylum and genus levels, respectively (Figure 6A,B). The contribution of soil pH to bacterial community composition was the greatest and the most significant (Table S5).

Spearman’s correlation analysis of major bacterial phyla and soil properties showed that the relative abundance of Chloroflexi was significantly negatively related to soil pH value, NH_4_^+^–N and NO_3_^−^–N contents. The abundance of Nitrospirae was positively correlated with soil pH value, SOM, NH_4_^+^–N and NO_3_^−^–N contents. Most phyla such as Verrucomicrobia, Gemmatimonadetes and Nitrospirae were positively linked with soil pH value. The abundance of Gemmatimonadetes and soil AP content, Patescibacteria and SOM content were significantly positive correlations (Figure 6A). For the genera (Figure 6B), Subgroup_2 and Acidothermus, which belonged to Acidobacteria and Actinobacteria, respectively, and their abundances were negatively related to soil pH value. HSB_OF53-F07 and Acidibacter showed strong negative correlations with most edaphic factors; they belong to Chloroflexi and Proteobacteria, respectively. The inverse relationship existed between the soil pH value and the abundances of Subgroup_2 (Acidobacteria) and Acidothermus (Actinobacteria).

## 4. Discussion

### 4.1. Variations in Soil Chemical Properties under Different Fertilization Treatments

It is widely known teak grows better in rich soils with a neutral and slightly alkaline pH [50,51]. Teak is a species that shows a strong preference for calcium and is sensitive to soil acidity in its growth process [39]. In this study, we found that the soil pH value increased after the combined application of organic and inorganic fertilizers, and soil pH was significantly higher in OCPF treatment than no fertilization treatment (*p* < 0.05, Table 1). The main reason could be attributed to the high calcium oxide content in the soil due to the addition of CaMgP fertilizer [52].

The increase in the soil pH value might also be related to the decomposition of soil organic matter [53]. A significant improvement in soil nutrient status characterized by organic matter, nitrogen and phosphorus enrichment was observed as a result of the mixed fertilization (Table 1). The application of organic−inorganic fertilizers could regulate soil aggregates and provide a source enriched in carbon and nitrogen for soil microorganisms, which promotes their activity and growth [22,54]. Correspondingly, bacterial taxa can mediate soil carbon cycling and effectively release more nutrients from organic matter [55,56]. The cumulation of soil nutrients was directly or indirectly influenced by bacterial communities, such as Gemmatimonadetes and Nitrospirae taxa, which are closely related to soil carbon degradation and nitrogen mineralization [57,58]. In this study, the increase of bacterial abundance in OCPF (Figure S3) might promote the level of soil carbon and nitrogen. Gemmatimonadetes taxa were identified as one of the biomarkers and keystone taxa in OCPF treatment (Figure 5 and Figure 6), which is directly related to the highest level of organic matter in this soil.

Soil pH may play a major role in transforming soil nutrient contents by changing bacterial taxa. Microbial communities exhibit poor growth in lower pH environments, and a relatively acidic condition usually inhibits both enzyme activity and overall cellular metabolism [59,60]. A previous study also found that the application of organic fertilizer would strengthen the activity of phosphatase and invertase [61]. Moreover, bacterial networks and interactions among taxa exhibited higher complexity after OCPF treatment, which had a positive impact on nutrient cycling and accumulation [62].

### 4.2. Response of Bacterial Community to Soil Chemical Properties

Fertilization had a positive impact on soil pH and nutrient levels, directly or indirectly influencing changes in bacterial community composition and abundance. In the present study, soil pH and organic matter were the main impact factors after fertilization in the teak plantation (Table S5), which appears consistent with the findings of Wang et al. [63] and Zhang et al. [64], who researched soil bacterial communities in response to organic fertilizer application. However, Zhao et al. [65] reported that soil nutrients (e.g., organic matter, total N and total P), rather than pH, showed a significant correlation with the majority of abundant taxa. This may be caused by different fertilization strategies. Our study found that the abundance of Chloroflexi and WPS-2 decreased with increasing soil pH value (Figure 6). This result suggested that acidic conditions are more suitable for the development of both types of bacteria. On the contrary, most bacterial phyla are obviously enriched as the soil pH value increases. For example, Verrucomicrobia, Latescibacteria, Nitrospirae and Gemmatimonadetes prefer neutral over acidic environments [66]. It may be possible that the degeneration or loss of metabolic function of these phyla in acidic soils occurs when the pH value is below a certain threshold [67]. Our study also found that the relative abundance of Gemmatimonadetes was positively associated with soil available phosphorus, which was also corroborated by the result that the eco-function of Gemmatimonadetes taxa was positively correlated with soil nutrients [57].

The application of organic fertilizer plays a substantial role in sustaining soil organic matter levels and simultaneously, the input of organic materials is also beneficial for the improvement of soil aggregate structure and available nutrients [68]. These changes further affect the distribution, abundance, activity and composition of the microbial community [2]. A clear negative relationship was observed between the relative abundance of Acidibacter (which belongs to Proteobacteria) and SOM content, which was inconsistent with the phenomenon that Proteobacteria taxa have a fast growth rate and are more likely to grow in nutrient-rich conditions [69]. This is potentially because of the site heterogeneity exhibited by bacterial taxa in soil, along with their substrate preferences, and the exact reasons for this inconsistency need further investigation.

### 4.3. Effects of Fertilization on Soil Bacterial Community Composition and Structure

Bacteria are the most abundant and diverse group of soil microorganisms, maintaining the stability and sustainability of the soil ecosystem in forests [70,71]. The decline in bacterial diversity may directly affect the stability of soil microbial communities [31]. Fertilization leads to changes in the structure of soil bacterial communities by changing the living environment of microorganisms. Previous studies found that urea or NPK fertilizer application inhibited the growth of microorganisms and showed a significant negative effect on bacterial diversity [72], while the addition of organic manure [73] or biological organic fertilizer [74] shaped a richer variety of soil bacteria. It was also reported that the diversity and richness of the bacterial community did not change significantly with either the application of organic fertilizer or mineral fertilizer [64,75]. In this study, the number of effective sequences showed a slight decline after fertilization (Table S3), and the alpha diversity of bacterial communities did not differ significantly between CK and fertilization treatments (Figure 1). Possible reasons for this result are the short duration of the fertilizer’s application or soil heterogeneity between treatments.

Determining the variations in soil bacterial composition between fertilization versus no fertilization treatment can effectively assess the effect of soil quality, health and fertilization on artificial forest ecosystems [76]. In the present study, although fertilization had no effect on the alpha diversity of bacteria (Figure 1), significant differences were revealed in bacterial community structure among the different treatments (*p* < 0.01, Figure S2). Acidobacteria and Proteobacteria were two of the most abundant phyla observed in the bacterial community, as shown in previous studies [77,78]. Both of them can properly enhance soil fertility and sustainability when they were enriched in soil [60]. It was reported that Acidobacteria were functionally diverse and contributed to the degradation of root exudates and litter to stabilize organic matter content in soil [79]. The activity of Acidobacteria taxa (such as Subgroup_2 and Candidatus_Solibacter) was more influenced by OCPF because the effect of soil pH may be stronger than that of organic matter, resulting in a decrease in the abundance of Acidobacteria (Table S4, Figure 6A). Firmicutes were the third most dominant bacterial phylum in the tested soils (Figure 1, Table S4), and were considered to be the copiotrophic taxa in previous reports [63,80,81]. The addition of effective nutrients through OCPF and CPF treatments might create a more favorable environment for Firmicute taxa multiplication, thus expanding the abundance of Firmicutes. Additionally, Gemmatimonadetes, Myxococcota and Actinobacteria under OCPF treatment were significantly enriched, which may be attributed to their ecological function related to the degradation capacity of organic matter [82,83]. Contrarily, the application of OCPF, OCF and CPF had significant side effects on the relative abundance of Chloroflexi, and the growth and development of this phylum taxa are likely to be limited by the accumulation of soil nutrients. This is similar to the results of Liu et al. [76] and Durand et al. [84], who indicated that Chloroflexi is slow-growing taxa that is classified as an oligotroph.

### 4.4. Network Patterns of Soil Bacterial Community

Co-occurrence network analysis provides evidence of the direct and indirect cooperation or composition relationships among microbial taxa [85]. The interaction among bacterial taxa is closely correlated with their ecological functions comprising nutrient metabolism associated with the C, N and P cycles [86,87]. The linkage density among species based on the network contributed to predicting ecosystem stability and complexity. This is because the network complexity is often dependent upon the number of edges and nodes within the network [28]. More network interactions and connectors (taxa) are fundamental to stabilize the microbial community structure [88].

Our study found that fertilization altered the relationship between bacterial species, and soil bacterial communities in different treatments exhibited different patterns of co-occurrence networks (Figure 5). The number of linkage edges and network degrees was increased in all fertilization treatments. The number of nodes and edges and the network density after OCPF were greater than in CK and other fertilization treatments (Table 2), indicating that interspecies relationships and community structures of bacteria were more complicated in the soil. The stronger interactions of bacterial taxa in OCPF treatment compared to CK possibly accelerated the metabolic activity of microorganisms and promoted the accumulation of soil nutrients. The higher content of soil organic matter and nitrate−nitrogen in OCPF also supported this result (Table 1). Yu et al. [49] also indicated that bacterial taxa within the complex network structure play a prominent role in promoting soil nutrient cycling in teak plantations. Moreover, the addition of fertilizers decreased the proportion of positive edges compared to no fertilization (Figure 5, Table 2), indirectly suggesting that soil effective nutrients aggravated competition and niche separation of bacterial species [89,90]. The lower nutrient environment allowed a large number of bacterial taxa to coexist, while high nutrient environments caused more negative interactions among species [91]. These opposing relationships inhibited each other and excluded more species in the community composition, resulting in a diminution of species diversity [18,28]. Network diameter, modularity coefficient and average path length based on OCPF treatment were relatively smaller than others (Figure 5). The result demonstrated that an intensive world network formed after OCPF treatment to rapidly respond to alterations in the soil microenvironment [92].

## 5. Conclusions

In this study, mixed fertilization with organic, NPK and phosphorus fertilizers significantly increased the content of soil organic matter and available nutrients and reduced soil acidity in young teak plantations. The changes in organic matter level and pH value in soil further altered the composition and network of the bacterial community. The relative abundance of Gemmatimonadetes and Myxococcota in OCPF treatment was clearly enriched, while it was decreased for Chloroflexi, Acidobacteria and Proteobacteria. The topological properties of the co-occurrence network showed that the nodes, edges, network density and average degree were the highest in OCPF, indicating that the bacterial community structure in this soil was more stable and complex. Moreover, soil pH and organic matter were closely associated with bacterial species and were regarded as the primary factors shaping bacterial community structure. Further research on the impact of keystone species within microorganisms is required to identify their contribution to nutrient cycling and to predict the coupling relationship between their function and forest productivity in plantation ecosystems.

## Figures and Tables

**Table 1:** Soil chemical properties of different fertilization treatments.

Treatment	pH	SOM (g·kg^−1^)	NH_4_^+^–N (mg·kg^−1^)	NO_3_^−^–N (mg·kg^−1^)	AP (mg·kg^−1^)
OCF	5.04 ± 0.09 ^a,b^	42.21 ± 0.94 ^a,b^	78.21 ± 3.01 ^a^	11.95 ± 0.33 ^a^	2.52 ± 0.19 ^a^
OPF	5.03 ± 0.08 ^a,b^	40.86 ± 2.12 ^a,b^	33.75 ± 2.66 ^c,d^	6.68 ± 0.05 ^c^	2.32 ± 0.24 ^a,b^
OCPF	5.30 ± 0.08 ^a^	43.99 ± 1.39 ^a^	48.21 ± 3.96 ^b,c^	11.97 ± 0.41 ^a^	2.13 ± 0.06 ^a,b^
CPF	5.20 ± 0.08 ^a,b^	37.43 ± 0.97 ^b^	51.35 ± 2.05 ^b^	10.22 ± 0.26 ^b^	2.35 ± 0.19 ^a,b^
CK	4.90 ± 0.08 ^b^	36.54 ± 1.17 ^b^	30.92 ± 2.27 ^d^	6.27 ± 0.44 ^c^	1.70 ± 0.11 ^b^
*p*-value	0.028	0.008	<0.001	<0.001	0.035

Notes: values are the means ± standard error (*n* = 4). OCF, application of organic fertilizer and NPK compound fertilizer; OPF, application of organic and CaMgP fertilizers; OCPF, application of organic, NPK and CaMgP fertilizers; CPF, application of NPK and CaMgP fertilizers; CK, no fertilization. SOM, soil organic matter; NH_4_^+^–N, ammonium nitrogen; NO_3_^−^–N, nitrate−nitrogen; AP, available phosphorus. Different lowercase letters indicate significant differences at a 0.05 significance level among treatments.

**Table 2:** Topological properties of bacterial communities’ co-occurrence network for different treatments in the teak plantation.

Topological Properties	OCF	OPF	OCPF	CPF	CK
Nodes	97	98	99	96	98
Edges	285	431	500	426	273
Positive edge proportion	59.30%	66.82%	62.80%	72.77%	73.26%
Negative edge proportion	40.70%	33.18%	32.70%	27.23%	26.74%
Network density	0.061	0.091	0.103	0.091	0.057
Average degree	5.876	8.796	10.101	8.784	5.315
Average clustering coefficient	0.621	0.637	0.600	0.648	0.541
Network diameter	14	11	11	14	15
Modularity	0.735	0.608	0.527	0.550	0.653
Average path length	6.030	3.954	4.165	5.788	5.315

## Data Availability

The data presented in this study are available in the NCBI Sequence Read Achieve database (accession number: PRJNA820355).

## Supplementary Materials

The following supporting information can be downloaded at: https://www.mdpi.com/2076-2607/10/5/958/s1, Table S1: Fertilizer input for each treatment; Table S2: Raw reads, effective tags, mean length and goods coverage of soil samples after sequencing; Table S3: The values (mean, *n* = 4) of raw reads, effective tags, mean length and goods coverage among the different groups; Table S4: The relative abundance (*n* = 4) of soil bacterial communities at dominant phylum and genus levels under different fertilization treatments; Table S5: Mantel tests of the correlation between bacterial abundance on ASV levels and soil properties, and the contributions of soil chemical properties to variation in soil bacterial taxa; Figure S1: The similarities and differences in the number of ASVs among the different treatments; Figure S2: Principal coordinate analysis (PCoA) and ANOSIM based on weighted unifrac of a bacterial community on ASV level for 20 soil samples from different fertilization treatments; Figure S3: The linear discriminant analysis (LDA) effect size (LEfSe) of soil bacterial biomarkers under different fertilization treatments. The values were significant (*p* < 0.05) when the LDA score was more than 2.

## References

[1] Eo J., Park K.-C. (2016). Long-term effects of imbalanced fertilization on the composition and diversity of soil bacterial community. Agric. Ecosyst. Environ..

[2] Han S., Delgado-Baquerizo M., Luo X., Liu Y., Van Nostrand J.D., Chen W., Zhou J., Huang Q. (2021). Soil aggregate size-dependent relationships between microbial functional diversity and multifunctionality. Soil Biol. Biochem..

[3] Guo J.H., Liu X.J., Zhang Y., Shen J.L., Han W.X., Zhang W.F., Christie P., Goulding K.W.T., Vitousek P.M., Zhang F.S. (2010). Significant Acidification in Major Chinese Croplands. Science.

[4] Bai Y.-C., Chang Y.-Y., Hussain M., Lu B., Zhang J.-P., Song X.-B., Lei X.-S., Pei D. (2020). Soil Chemical and Microbiological Properties Are Changed by Long-Term Chemical Fertilizers That Limit Ecosystem Functioning. Microorganisms.

[5] Allison S.D., Martiny J.B.H. (2008). Resistance, resilience, and redundancy in microbial communities. Proc. Natl. Acad. Sci. USA.

[6] Zeng J., Liu X., Song L., Lin X., Zhang H., Shen C., Chu H. (2016). Nitrogen fertilization directly affects soil bacterial diversity and indirectly affects bacterial community composition. Soil Biol. Biochem..

[7] Zhou J., Jiang X., Wei D., Zhao B.S., Ma M.C., Chen S.F., Cao F.M., Shen D.L., Guan D.W., Li J. (2017). Consistent effects of nitrogen fertilization on soil bacterial communities in black soils for two crop seasons in China. Sci. Rep..

[8] Wang J., Song Y., Ma T., Raza W., Li J., Howland J.G., Huang Q., Shen Q. (2017). Impacts of inorganic and organic fertilization treatments on bacterial and fungal communities in a paddy soil. Appl. Soil Ecol..

[9] Li R., Tao R., Ling N., Chu G. (2017). Chemical, organic and bio-fertilizer management practices effect on soil physicochemical property and antagonistic bacteria abundance of a cotton field: Implications for soil biological quality. Soil Tillage Res..

[10] Sihi D., Dari B., Sharma D.K., Pathak H., Nain L., Sharma O.P. (2017). Evaluation of soil health in organic vs. conventional farming of basmati rice in North India. J. Plant Nutr. Soil Sci..

[11] Sheoran H.S., Kakar R., Kumar N. (2019). Seema impact of organic and conventional farming practices on soil quality: A global review. Appl. Ecol. Environ. Res..

[12] Muhammad Q., Huang J., Waqas A., Li D.C., Liu S.J., Zhang L., Cai A.D., Liu L.S., Xu Y.M., Gao J.S. (2020). Yield sustainability, soil organic carbon sequestration and nutrients balance under long-term combined application of manure and inorganic fertilizers in acidic paddy soil. Soil Till. Res..

[13] Wei M., Hu G., Wang H., Bai E., Lou Y., Zhang A., Zhuge Y. (2017). 35 years of manure and chemical fertilizer application alters soil microbial community composition in a Fluvo-aquic soil in Northern China. Eur. J. Soil Biol..

[14] Mei N., Zhang X., Wang X., Peng C., Gao H., Zhu P., Gu Y. (2021). Effects of 40 years applications of inorganic and organic fertilization on soil bacterial community in a maize agroecosystem in northeast China. Eur. J. Agron..

[15] Falkowski P.G., Fenchel T., Delong E.F. (2008). The Microbial Engines That Drive Earth’s Biogeochemical Cycles. Science.

[16] Hu Y., Zhang Z., Huang L., Qi Q., Liu L., Zhao Y., Wang Z., Zhou H., Lv X., Mao Z. (2019). Shifts in soil microbial community functional gene structure across a 61-year desert revegetation chronosequence. Geoderma.

[17] Grzyb A., Wolna-Maruwka A., Niewiadomska A. (2020). Environmental Factors Affecting the Mineralization of Crop Residues. Agronomy.

[18] Wu H., Cai A., Xing T., Huai S., Zhu P., Xu M., Lu C. (2021). Fertilization enhances mineralization of soil carbon and nitrogen pools by regulating the bacterial community and biomass. J. Soils Sediments.

[19] Wang Q., Jiang X., Guan D., Wei D., Zhao B., Ma M., Chen S., Li L., Cao F., Li J. (2018). Long-term fertilization changes bacterial diversity and bacterial communities in the maize rhizosphere of Chinese Mollisols. Appl. Soil Ecol..

[20] Bissett A., Richardson A.E., Baker G., Thrall P.H. (2011). Long-term land use effects on soil microbial community structure and function. Appl. Soil Ecol..

[21] Hartman K., van der Heijden M.G., Wittwer R.A., Banerjee S., Walser J.C., Schlaeppi K. (2018). Cropping practices manipulate abundance patterns of root and soil microbiome members paving the way to smart farming. Microbiome.

[22] Wu L.P., Wang Y.D., Zhang S.R., Wei W.L., Kuzyakov Y., Ding X.D. (2021). Fertilization effects on microbial community compo-sition and aggregate formation in saline-alkaline soil. Plant Soil.

[23] Li X., Jiao X., Wang H., Wang G. (2021). Organic-inorganic combined fertilization alters reclaimed soil bacterial communities in an opencast coal mine area and improves soil quality. Arab. J. Geosci..

[24] Francioli D., Schulz E., Lentendu G., Wubet T., Buscot F., Reitz T. (2016). Mineral vs. Organic Amendments: Microbial Community Structure, Activity and Abundance of Agriculturally Relevant Microbes Are Driven by Long-Term Fertilization Strategies. Front. Microbiol..

[25] Ye C., Huang S., Sha C., Wu J., Cui C., Su J., Ruan J., Tan J., Tang H., Xue J. (2022). Changes of bacterial community in arable soil after short-term application of fresh manures and organic fertilizer. Environ. Technol..

[26] Tao R., Liang Y., Wakelin S., Chu G. (2015). Supplementing chemical fertilizer with an organic component increases soil biological function and quality. Appl. Soil Ecol..

[27] Wolna-Maruwka A., Piechota T., Niewiadomska A., Kamiński A., Kayzer D., Grzyb A., Pilarska A.A. (2021). The effect of bio-char-based organic amendments on the structure of soil bacterial community and yield of maize (*Zea mays* L.). Agronomy.

[28] Wagg C., Schlaeppi K., Banerjee S., Kuramae E.E., Van Der Heijden M.G.A. (2019). Fungal-bacterial diversity and microbiome complexity predict ecosystem functioning. Nat. Commun..

[29] Ma B., Lv X., Cai Y., Chang S.X., Dyck M. (2018). Liming does not counteract the influence of long-term fertilization on soil bacterial community structure and its co-occurrence pattern. Soil Biol. Biochem..

[30] Yao T., Chen R., Zhang J., Feng Y., Huang M., Lin X. (2020). Divergent patterns of microbial community composition shift under two fertilization regimes revealed by responding species. Appl. Soil Ecol..

[31] Xu Y., Li C., Zhu Y., Wang Z., Zhu W., Wu L., Du A. (2022). The shifts in soil microbial community and association network induced by successive planting of *Eucalyptus* plantations. For. Ecol. Manag..

[32] Muneer M.A., Hou W., Li J., Huang X., Kayani M.U.R., Cai Y., Yang W., Wu L., Ji B., Zheng C. (2022). Soil pH: A key edaphic factor regulating distribution and functions of bacterial community along vertical soil profiles in red soil of pomelo orchard. BMC Microbiol..

[33] Zhang Y., Shen H., He X., Thomas B., Lupwayi N., Hao X., Thomas M.C., Shi X. (2017). Fertilization Shapes Bacterial Community Structure by Alteration of Soil pH. Front. Microbiol..

[34] Brennan E.B., Acosta-Martinez V. (2017). Cover cropping frequency is the main driver of soil microbial changes during six years of organic vegetable production. Soil Biol. Biochem..

[35] Li W.X., Zhang F.Y., Cui G.H., Wang Y.N., Yang J.G., Cheng H.C., Liu H.W., Zhang L.P. (2021). Effects of bio-organic fertilizer on soil fertility microbial community composition, and potato growth. Sci. Asia.

[36] Huang G., Liang K., Zhou Z., Yang G., Muralidharan E.M. (2019). Variation in Photosynthetic Traits and Correlation with Growth in Teak (*Tectona grandis* Linn.) Clones. Forests.

[37] Masilamani P., Albert V.A., Venkatesan S., Janaki P. (2020). Effect of phosphorus application on growth and stump quality of teak (*Tectona grandis* L.f.) seedlings. Madras Agric. J..

[38] Balam-Che M., Gomez-Guerrero A., Vargas-Hernández J.V., Aldrete A., Obrador-Olán J.J. (2015). Initial fertilization in commercial plantations of teak (*Tectona grandis* L.f.) in southeast México. Rev. Fitotec. Mex..

[39] Wehr J.B., Blamey F.P.C., Smith T.E., Menzies N.W. (2017). Growth and physiological responses of teak (*Tectona grandis* L.f.) clones to Ca, H and Al stress in solution and acid soils. New For..

[40] Wiratama A. (2015). The Fertilization Giving Effect Basic Plant Growth of Teak (Tectona grandis) Age to 6 Months in the Village Um-Bulmartani, Ngemplak District, Sleman Regeny.

[41] Dong W.-Y., Zhang X.-Y., Dai X.-Q., Fu X.-L., Yang F.-T., Liu X.-Y., Sun X.-M., Wen X.-F., Schaeffer S. (2014). Changes in soil microbial community composition in response to fertilization of paddy soils in subtropical China. Appl. Soil Ecol..

[42] Wu L., Li Z., Li J., Khan M.A., Huang W., Zhang Z., Lin W. (2013). Assessment of shifts in microbial community structure and catabolic diversity in response to *Rehmannia glutinosa* monoculture. Appl. Soil Ecol..

[43] Reijonen I., Metzler M., Hartikainen H. (2016). Impact of soil pH and organic matter on the chemical bioavailability of vanadium species: The underlying basis for risk assessment. Environ. Pollut..

[44] Liu X., Shi Y., Kong L., Tong L., Cao H., Zhou H., Lv Y. (2022). Long-Term Application of Bio-Compost Increased Soil Microbial Community Diversity and Altered Its Composition and Network. Microorganisms.

[45] Magoč T., Salzberg S.L. (2011). FLASH: Fast length adjustment of short reads to improve genome assemblies. Bioinformatics.

[46] Haas B.J., Gevers D., Earl A.M., Feldgarden M., Ward D.V., Giannoukos G., Ciulla D., Tabbaa D., Highlander S.K., Sodergren E. (2011). Chimeric 16S rRNA sequence formation and detection in Sanger and 454-pyrosequenced PCR amplicons. Genome Res..

[47] Li M.J., Shao D.T., Zhou J.C., Gu J.H., Qin J.J., Chen W., Wei W.Q. (2020). Signatures within esophageal microbiota with pro-gression of esophageal squamous cell carcinoma. Chin. J. Cancer. Res..

[48] Li S., Wu F. (2018). Diversity and Co-occurrence Patterns of Soil Bacterial and Fungal Communities in Seven Intercropping Systems. Front. Microbiol..

[49] Yu Z., Liang K., Huang G., Wang X., Lin M., Chen Y., Zhou Z. (2021). Soil Bacterial Community Shifts Are Driven by Soil Nutrient Availability along a Teak Plantation Chronosequence in Tropical Forests in China. Biology.

[50] Fernández-Moya J., Alvarado A., Mata R., Thiele H., Segura J.M., Vaides E., Miguel-Ayanz A.S., Marchamalo-Sacristán M. (2015). Soil fertility characterisation of teak (*Tectona grandis* L.f.) plantations in Central America. Soil Res..

[51] Zhou Z., Liu S., Liang K., Ma H., Huang G. (2016). Growth and mineral nutrient analysis of teak (*Tectona grandis*) grown on acidic soils in south China. J. For. Res..

[52] Mkhonza N.P., Buthelezi-Dube N.N., Muchaonyerwa P. (2020). Effects of lime application on nitrogen and phosphorus availability in humic soils. Sci. Rep..

[53] Hu X., Liu J., Wei D., Zhu P., Cui X., Zhou B., Chen X., Jin J., Liu X., Wang G. (2018). Soil Bacterial Communities Under Different Long-Term Fertilization Regimes in Three Locations Across the Black Soil Region of Northeast China. Pedosphere.

[54] Muhammad Q., Huang J., Waqas A., Muhammad A., Li D.C., Zulqarnain H.K., Gao J.S., Liu S.J., Zhang H.M. (2021). Linkages between ecoenzymatic stoichiometry and microbial community structure under long-term fertilization in paddy soil: A case study in China. Appl. Soil Ecol..

[55] Frey S., Six J., Elliott E. (2003). Reciprocal transfer of carbon and nitrogen by decomposer fungi at the soil–litter interface. Soil Biol. Biochem..

[56] Terrazas R.A., Giles C., Paterson E., Robertson-Albertyn S., Cesco S., Mimmo T., Pii Y., Bulgarelli D. (2016). Chapter one-plant-microbiota interactions as a driver of the mineral turnover in the rhizosphere. Adv. Appl. Microbiol..

[57] Mujakić I., Piwosz K., Koblek M. (2022). Phylum Gemmatimonadetes and its role in the environment. Microorganisms.

[58] Castellano-Hinojosa A., Strauss S.L., Gonzálea- López J., Bedmar E.J. (2021). Changes in the diversity and predicted functional composition of the bulk and rhizosphere soil bacterial microbiomes of tomoto and common bean after inorganic N-fertilization. Rhizosphere.

[59] Beales N. (2004). Adaptation of Microorganisms to Cold Temperatures, Weak Acid Preservatives, Low pH, and Osmotic Stress: A Review. Compr. Rev. Food Sci. Food Saf..

[60] Rousk J., Brookes P.C., Bååth E. (2009). Contrasting Soil pH Effects on Fungal and Bacterial Growth Suggest Functional Redundancy in Carbon Mineralization. Appl. Environ. Microbiol..

[61] Marcote I., Hernández T., García C., Polo A. (2001). Influence of one or two successive annual applications of organic fertilisers on the enzyme activity of a soil under barley cultivation. Bioresour. Technol..

[62] Dahlawi S., Naeem A., Rengel Z., Naidu R. (2018). Biochar application for the remediation of salt-affected soils: Challenges and opportunities. Sci. Total Environ..

[63] Wang X., Li Y., Wei Y., Meng H., Cao Y., Lead J., Hong J. (2020). Effects of fertilization and reclamation time on soil bacterial communities in coal mining subsidence areas. Sci. Total Environ..

[64] Zhang S.N., Sun L.T., Wang Y., Fan K., Xu Q.S., Li Y.S., Ma Q.P., Wang J.G., Ren W.M., Ding Z.T. (2020). Cow manure appli-cation effectively regulates the soil bacterial community in tea plantation. BMC Microbiol..

[65] Zhao J., Tian N., Yong L., Xiong W., Ran W. (2014). Responses of Bacterial Communities in Arable Soils in a Rice-Wheat Cropping System to Different Fertilizer Regimes and Sampling Times. PLoS ONE.

[66] DeBruyn J.M., Nixon L.T., Fawaz M.N., Johnson A.M., Radosevich M. (2011). Global Biogeography and Quantitative Seasonal Dynamics of Gemmatimonadetes in Soil. Appl. Environ. Microbiol..

[67] Rousk J., Bååth E., Brookes P.C., Lauber C.L., Lozupone C., Caporaso J.G., Knight R., Fierer N. (2010). Soil bacterial and fungal communities across a pH gradient in an arable soil. ISME J..

[68] Bansal S., Yin X.H., Sykes V., Lee J., Jagadamma S. (2021). Soil aggregate-associated organic carbon and nitrogen response to long-term no-till crop rotation, cover crop, and manure application. Soil Sci. Soc. Am. J..

[69] Dai Z., Su W., Chen H., Barberán A., Zhao H., Yu M., Yu L., Brookes P.C., Schadt C.W., Chang S.X. (2018). Long-term nitrogen fertilization decreases bacterial diversity and favors the growth of *Actinobacteria* and *Proteobacteria* in agro-ecosystems across the globe. Glob. Chang. Biol..

[70] Gans J., Wolinsky M., Dunbar J. (2005). Computational Improvements Reveal Great Bacterial Diversity and High Metal Toxicity in Soil. Science.

[71] Xue L., Ren H., Brodribb T.J., Wang J., Yao X., Li S. (2021). Long term effects of management practice intensification on soil microbial community structure and co-occurrence network in a non-timber plantation. For. Ecol. Manag..

[72] Wang F., Chen S., Qin S., Sun R., Zhang Y., Wang S., Hu C., Hu H., Liu B. (2021). Long-term nitrogen fertilization alters microbial community structure and denitrifier abundance in the deep vadose zone. J. Soils Sediments.

[73] Qiu S.-L., Wang L.-M., Huang D.-F., Lin X.-J. (2014). Effects of fertilization regimes on tea yields, soil fertility, and soil microbial diversity. Chil. J. Agric. Res..

[74] Hamm A.C., Tenuta M., Krause D.O., Ominski K.H., Tkachuk V.L., Flaten D.N. (2016). Bacterial communities of an agricultural soil amended with solid pig and dairy manures, and urea fertilizer. Appl. Soil Ecol..

[75] Wu X., Zhang T., Zhao J., Wang L., Yang D., Li G., Xiu W. (2020). Variation of Soil Bacterial and Fungal Communities from Fluvo-Aquic Soil Under Chemical Fertilizer Reduction Combined with Organic Materials in North China Plain. J. Soil Sci. Plant Nutr..

[76] Liu S., Li P., Van Zwieten L., Tu J., Gan W., Lu S., Wang H., Wu L. (2021). Edaphic variables influence soil bacterial structure under successive fertilization of Paulownia plantation substituting native vegetation. J. Soils Sediments.

[77] Fierer N. (2017). Embracing the unknown: Disentangling the complexities of the soil microbiome. Nat. Rev. Genet..

[78] Rao D., Meng F., Yan X., Zhang M., Yao X., Kim K.S., Zhao J., Qiu Q., Xie F., Zhang W. (2021). Changes in Soil Microbial Activity, Bacterial Community Composition and Function in a Long-Term Continuous Soybean Cropping System After Corn Insertion and Fertilization. Front. Microbiol..

[79] Niu Y., Zhang M., Bai S.H., Xu Z., Liu Y., Chen F., Guo X., Luo H., Wang S., Xie J. (2020). Successive mineral nitrogen or phosphorus fertilization alone significantly altered bacterial community rather than bacterial biomass in plantation soil. Appl. Microbiol. Biotechnol..

[80] Fierer N., Bradford M.A., Jackson R.B. (2007). Toward an ecological classification of soil bacteria. Ecology.

[81] Lennon J.T., Jones S.E. (2011). Microbial seed banks: The ecological and evolutionary implications of dormancy. Nat. Rev. Genet..

[82] Reddy P.P. (2014). Plant Growth Promoting Rhizobacteria for Horticultural Crop Protection.

[83] Liu R., Wang Z., Wang L., Li Z., Fang J., Wei X., Wei W., Cao J., Wei Y., Xie Z. (2020). Bulk and Active Sediment Prokaryotic Communities in the Mariana and Mussau Trenches. Front. Microbiol..

[84] Durand A., Maillard F., Alvarez-Lopez V., Guinchard S., Bertheau C., Valot B., Blaudez D., Chalot M. (2018). Bacterial diversity associated with poplar trees grown on a Hg-contaminated site: Community characterization and isolation of Hg-resistant plant growth-promoting bacteria. Sci. Total Environ..

[85] Fournier B., Dos Santos S.P., Gustavsen J.A., Imfeld G., Lamy F., Mitchell E.A., Mota M., Noll D., Planchamp C., Heger T.J. (2020). Impact of a synthetic fungicide (fosetyl-Al and propamocarb-hydrochloride) and a biopesticide (*Clonostachys rosea*) on soil bacterial, fungal, and protist communities. Sci. Total Environ..

[86] Kyei-Boahen S., Slinkard A., Walley F. (2002). Isotopic fractionation during N2 fixation by chickpea. Soil Biol. Biochem..

[87] Banerjee S., Kirkby C.A., Schmutter D., Bissett A., Kirkegaard J., Richardson A.E. (2016). Network analysis reveals functional redundancy and keystone taxa amongst bacterial and fungal communities during organic matter decomposition in an arable soil. Soil Biol. Biochem..

[88] Morriën E., Hannula S.E., Snoek B., Helmsing N.R., Zweers H., De Hollander M., Soto R.L., Bouffaud M.-L., Buée M., Dimmers W. (2017). Soil networks become more connected and take up more carbon as nature restoration progresses. Nat. Commun..

[89] Yu J., Deem L.M., Crow S.E., Deenik J.L., Penton C.R. (2018). Biochar application influences microbial assemblage complexity and composition due to soil and bioenergy crop type interactions. Soil Biol. Biochem..

[90] Ge Z., Li S.Y., Bol R., Zhu P., Peng C., An T.T., Cheng N., Liu X., Li T.Y., Xu Z.Q. (2021). Differential long-term fertilization alters residue-derived labile organic carbon fractions and microbial community during straw residue decomposition. Soil Till. Res..

[91] Ratzke C., Barrere J., Gore J. (2020). Strength of species interactions determines biodiversity and stability in microbial communities. Nat. Ecol. Evol..

[92] Zheng W., Zhao Z., Gong Q., Zhai B., Li Z. (2018). Responses of fungal–bacterial community and network to organic inputs vary among different spatial habitats in soil. Soil Biol. Biochem..

