# Supplementary material for: The Application of Mixed Organic and Inorganic Fertilizers Drives Soil Nutrient and Bacterial Community Changes in Teak Plantations
Source: Microorganisms. 2022 May 2;10(5):958. doi: 10.3390/microorganisms10050958 (PMC9145699; doi:10.3390/microorganisms10050958)
Supplement: Supplementary file 1 [file microorganisms-10-00958-s001.zip › microorganisms-1678155-supplementary.pdf]

Table S1. Fertilizer input for each treatment.

Treatment	Organic fertilizer ($t{\cdot}ha^{-1}{\cdot}yr^{-1}$)	NPK compound fertilizer ($t{\cdot}ha^{-1}{\cdot}yr^{-1}$)	CaMgP fertilizer ($t{\cdot}ha^{-1}{\cdot}yr^{-1}$)
OCF	1.056	1.584	0
OPF	2.112	0	1.584
OCPF	3.168	1.056	0.528
CPF	0	0.528	1.056
CK	0	0	0

CK, control; CPF, application of NPK compound fertilizer and CaMgP fertilizer; OCF, application of organic fertilizer and NPK compound fertilizer; OPF, application of organic fertilizer and CaMgP fertilizer; OCPF, application of organic fertilizer, NPK compound fertilizer and CaMgP fertilizer.

Table S2. Raw reads, effective tags, mean length and goods coverage of soil samples after sequencing.

Sample	Raw reads	Effective tags	Mean length	Goods coverage
OCF1	115,266	72,411	412	0.999
OCF2	101,547	58,380	412	0.999
OCF3	112,686	63,548	411	1.000
OCF4	103,263	53,149	411	1.000
OPF1	109,408	69,050	412	1.000
OPF2	118,649	72,966	412	0.999
OPF3	112,073	68,507	412	0.999
OPF4	110,000	55,847	411	1.000
OCPF1	112,547	59,060	414	1.000
OCPF2	104,880	59,079	413	1.000
OCPF3	115,653	66,036	415	1.000
OCPF4	116,763	58,022	412	1.000
CPF1	117,507	66,260	413	1.000
CPF2	112,464	55,235	415	1.000
CPF3	102,871	48,125	416	1.000
CPF4	104,094	55,101	416	1.000
CK1	104,611	62,564	411	1.000
CK2	116,838	66,979	411	1.000
CK3	106,942	84,966	412	0.999
CK4	117,771	70,918	412	0.999

Table S3. The values (mean, $n = 4$) of raw reads, effective tags, mean length and goods coverage among the different groups.

Treatment	Raw data	Effective tags	Mean length	Goods coverage (%)
OCF	108,191	61,872	411.5 b	99.95
OPF	112,533	66,593	411.8 b	99.95
OCPF	112,461	60,549	413.5 ab	100.0
CPF	109,234	56,180	415.0 a	100.0
CK	111,541	71,357	411.5 b	99.95
p value	0.791	0.112	0.000	0.293

Note: Different lowercase letters indicate significant differences at a 0.05 significance level among treatments.

Table S4. The relative abundance ($n = 4$) of soil bacterial communities at dominant phylum and genus levels under different fertilization treatments

Phylum	OCF	OPF	OCPF	CPF	CK	Genus	OCF	OPF	OCPF	CPF	CK
Acidobacteria	45.64	44.28	39.81	32.79	42.94	Subgroup_2	17.49	13.66	9.35	9.51	15.91
Proteobacteria	20.28	23.27	18.24	19.52	21.02	Candidatus_Solibacter	4.30	3.19	2.87	2.70	4.20
Firmicutes	7.55	6.28	10.29	11.12	6.61	Bifidobacterium	1.58	1.40	1.90	1.72	1.11
Actinobacteria	7.25	6.94	7.18	8.68	6.94	ADurb.Bin063-1	1.52	1.54	1.80	3.07	1.29
Chloroflexi	5.27	5.53	4.73	4.33	10.10	Acidothermus	2.04	1.93	1.09	1.38	2.64
Verrucomicrobia	2.99	4.23	4.17	5.70	3.82	JG30-KF-AS9	1.64	0.94	0.78	1.29	1.46
Gemmatimonadetes	2.87	3.21	3.90	3.59	1.77	Faecalibacterium	1.07	1.00	1.53	1.48	0.77
Bacteroidetes	1.44	2.26	2.72	3.76	2.20	Bradyrhizobium	1.55	2.06	1.34	1.35	1.42
Myxococcota	1.70	1.45	2.43	3.04	1.16	Candidatus_Udaeobacter	0.76	1.24	1.40	1.32	1.32
						Candidatus_Koribacter	1.40	1.38	1.14	1.01	0.92
						Bryobacter	1.39	1.19	1.15	1.28	1.62

Table S5. Mantel tests of the correlation between bacterial abundance on ASV levels and soil properties, and the contributions of soil chemical properties to variation in soil bacterial taxa (RDA analysis).

Variables	Mantel tests		Explanatory (%)	
	r value	P value	Phylum	Genus
pH	0.326	0.005**	7.8	33.2
SOM	0.249	0.026*	7.3	1.0
NH_4^+–N	0.014	0.429	2.2	5.7
NO_3^-–N	0.042	0.241	4.7	1.8
AP	0.035	0.358	1.4	1.1

SOM, soil organic matter; NH_4^+–N, ammonium nitrogen; NO_3^-–N, nitrate–nitrogen; AP, soil available phosphorus.

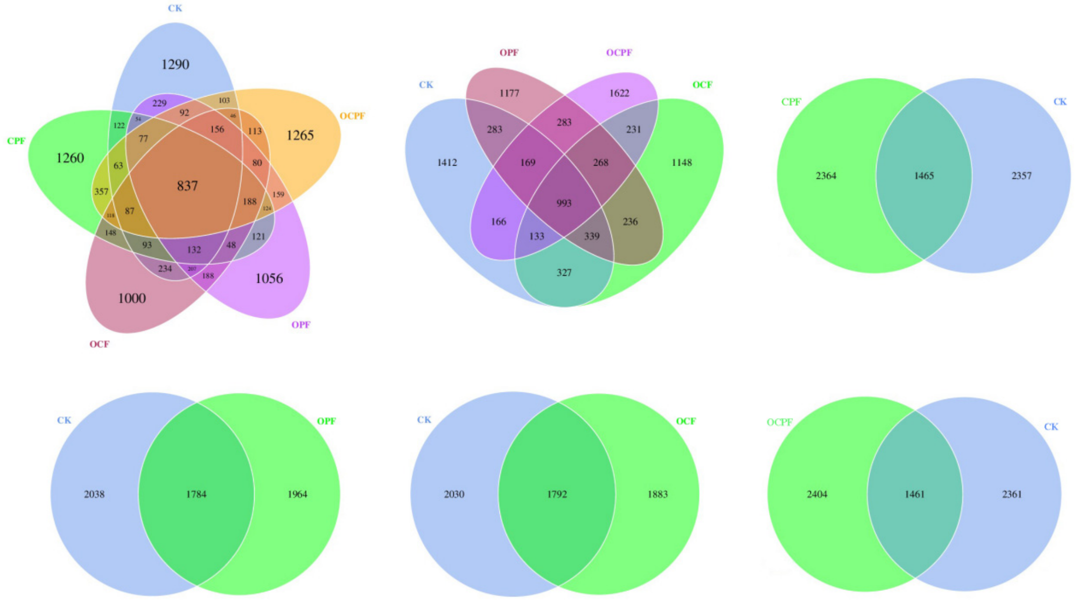

Figure S1. The similarities and differences in the number of ASVs among the different treatments.

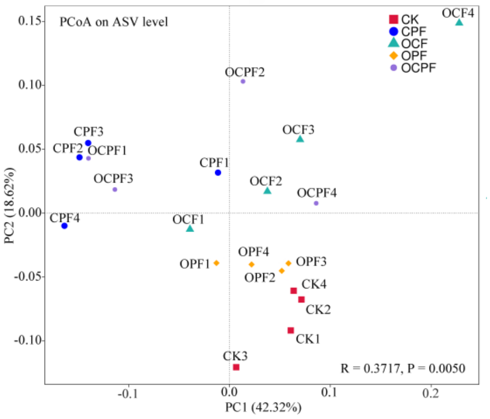

Figure S2. Principal coordinate analysis (PCoA) and ANOSIM based on weighted unifrac of a bacterial community on ASV level for 20 soil samples from different fertilization treatments.

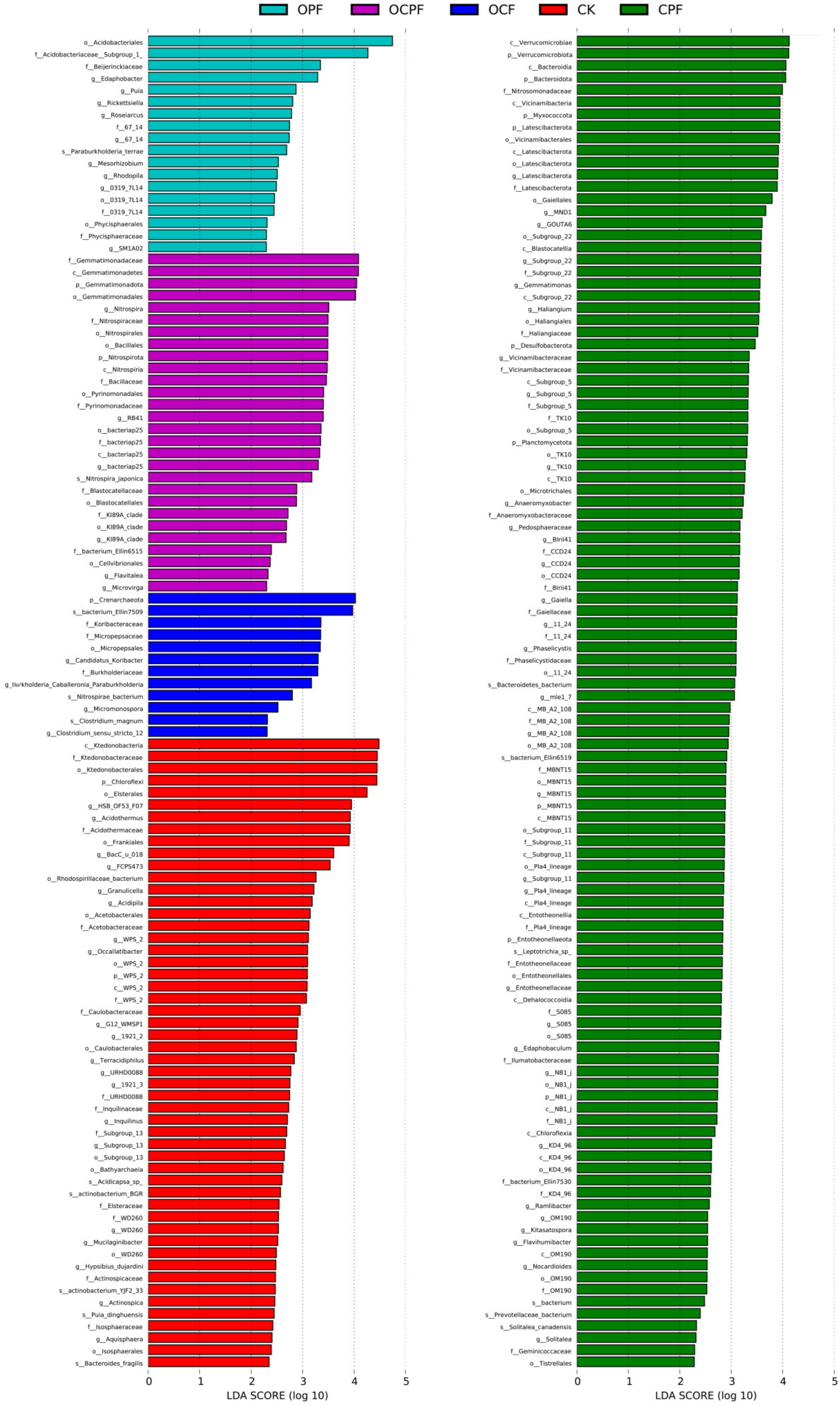
OPF
OCPF
OCF
CK
CPF
o__Acidobacteriales
f__Acidobacteriaceae__Subgroup_1_
f__Beijerinckiaceae
g__Edaphobacter
g__Puia
g__Rickettsiella
g__Roseiarcus
f__67_14
g__67_14
s__Paraburkholderia_terrae
g__Mesorhizobium
g__Rhodopila
g__0319_7L14
o__0319_7L14
f__0319_7L14
o__Phycisphaerales
f__Phycisphaeraceae
g__SM1A02
f__Gemmatimonadaceae
c__Gemmatimonadetes
p__Gemmatimonadota
o__Gemmatimonadales
g__Nitrospira
f__Nitrospiraceae
o__Nitrospirales
o__Bacillales
p__Nitrospirota
c__Nitrospiria
f__Bacillaceae
o__Pyrinomonadales
f__Pyrinomonadaceae
g__RB41
o__bacteriap25
f__bacteriap25
c__bacteriap25
g__bacteriap25
s__Nitrospira_japonica
f__Blastocatellaceae
o__Blastocatellales
f__KI89A_clade
o__KI89A_clade
g__KI89A_clade
f__bacterium_Ellin6515
o__Cellvibrionales
g__Flavitalea
g__Microvirga
p__Crenarchaeota
s__bacterium_Ellin7509
f__Koribacteraceae
f__Micropepsaceae
o__Micropepsales
g__Candidatus_Koribacter
f__Burkholderiaceae
g__Burkholderia_Caballeronia_Paraburkholderia
s__Nitrospirae_bacterium
g__Micromonospora
s__Clostridium_magnum
g__Clostridium_sensu_stricto_12
c__Ktedonobacteria
f__Ktedonobacteraceae
o__Ktedonobacterales
p__Chloroflexi
o__Elsterales
g__HSB_OF53_F07
g__Acidothermus
f__Acidothermaceae
o__Frankiales
g__BacC_u_018
g__FCPS473
o__Rhodospirillaceae_bacterium
g__Granulicella
g__Acidipila
o__Acetobacterales
f__Acetobacteraceae
g__WPS_2
g__Occallatibacter
o__WPS_2
p__WPS_2
c__WPS_2
f__WPS_2
f__Caulobacteraceae
g__G12_WMSP1
g__1921_2
o__Caulobacterales
g__Terracidiphilus
g__URHD0088
g__1921_3
f__URHD0088
f__Inquilinaceae
g__Inquilinus
f__Subgroup_13
g__Subgroup_13
o__Subgroup_13
o__Bathyarchaeia
s__Acidicapsa_sp_
s__actinobacterium_BGR
f__Elsteraceae
f__WD260
g__WD260
g__Mucilaginibacter
o__WD260
g__Hypsibius_dujardini
f__Actinospicaceae
s__actinobacterium_YJF2_33
g__Actinospica
s__Puia_dinghuensis
f__Isosphaeraceae
g__Aquisphaera
o__Isosphaerales
s__Bacteroides_fragilis
0
1
2
3
4
5
LDA SCORE (log 10)
c__Verrucomicrobiae
p__Verrucomicrobiota
c__Bacteroidia
p__Bacteroidota
f__Nitrosomonadaceae
c__Vicinamibacteria
p__Myxococcota
p__Latescibacterota
o__Vicinamibacterales
c__Latescibacterota
o__Latescibacterota
g__Latescibacterota
f__Latescibacterota
o__Gaiellales
g__MND1
g__GOUTA6
o__Subgroup_22
c__Blastocatellia
g__Subgroup_22
f__Subgroup_22
g__Gemmatimonas
c__Subgroup_22
g__Haliangium
o__Haliangiales
f__Haliangiaceae
p__Desulfobacterota
g__Vicinamibacteraceae
f__Vicinamibacteraceae
c__Subgroup_5
g__Subgroup_5
f__Subgroup_5
f__TK10
o__Subgroup_5
p__Planctomycetota
o__TK10
g__TK10
c__TK10
o__Microtrichales
g__Anaeromyxobacter
f__Anaeromyxobacteraceae
g__Pedosphaeraceae
g__Blrii41
f__CCD24
g__CCD24
o__CCD24
f__Blrii41
g__Gaiella
f__Gaiellaceae
g__11_24
f__11_24
g__Phaselicystis
f__Phaselicystidaceae
o__11_24
s__Bacteroidetes_bacterium
g__mle1_7
c__MB_A2_108
f__MB_A2_108
g__MB_A2_108
o__MB_A2_108
s__bacterium_Ellin6519
f__MBNT15
o__MBNT15
g__MBNT15
p__MBNT15
c__MBNT15
o__Subgroup_11
f__Subgroup_11
c__Subgroup_11
o__Pla4_lineage
g__Subgroup_11
g__Pla4_lineage
c__Pla4_lineage
c__Entotheonellia
f__Pla4_lineage
p__Entotheonellaeota
s__Leptotrichia_sp_
f__Entotheonellaceae
o__Entotheonellales
g__Entotheonellaceae
c__Dehalococcoidia
f__S085
g__S085
o__S085
g__Edaphobaculum
f__Ilumatobacteraceae
g__NB1_j
o__NB1_j
p__NB1_j
c__NB1_j
f__NB1_j
c__Chloroflexia
g__KD4_96
c__KD4_96
o__KD4_96
f__bacterium_Ellin7530
f__KD4_96
g__Ramlibacter
g__OM190
g__Kitasatospora
g__Flavihumibacter
c__OM190
g__Nocardioides
o__OM190
f__OM190
s__bacterium
s__Prevotellaceae_bacterium
s__Solitalea_canadensis
g__Solitalea
f__Geminicoccaceae
o__Tistrellales
0
1
2
3
4
5
LDA SCORE (log 10)

Figure S3. The linear discriminant analysis (LDA) effect size (LEfSe) of soil bacterial biomarkers under different fertilization treatments. The values were significant ($p < 0.05$) when the LDA score was more than 2.
